# Inversion of Winter Wheat Growth Parameters and Yield Under Different Water Treatments Based on UAV Multispectral Remote Sensing

**DOI:** 10.3389/fpls.2021.609876

**Published:** 2021-05-20

**Authors:** Xin Han, Zheng Wei, He Chen, Baozhong Zhang, Yinong Li, Taisheng Du

**Affiliations:** ^1^State Key Laboratory of Simulation and Regulation of Water Cycle in River Basin, China Institute of Water Resources and Hydropower Research, Beijing, China; ^2^College of Water Conservancy and Civil Engineering, China Agricultural University, Beijing, China; ^3^National Center of Efficient Irrigation Engineering and Technology Research, Beijing, China

**Keywords:** vegetation index, growth parameters, yield, optimal estimation method, different water treatments

## Abstract

In recent years, the unmanned aerial vehicle (UAV) remote sensing system has been rapidly developed and applied in accurate estimation of crop parameters and yield at farm scale. To develop the major contribution of UAV multispectral images in predicting winter wheat leaf area index (LAI), chlorophyll content (called soil and plant analyzer development [SPAD]), and yield under different water treatments (low water level, medium water level, and high water level), vegetation indices (VIs) originating from UAV multispectral images were used during key winter wheat growth stages. The estimation performances of the models (linear regression, quadratic polynomial regression, and exponential and multiple linear regression models) on the basis of VIs were compared to get the optimal prediction method of crop parameters and yield. Results showed that LAI and SPAD derived from VIs both had high correlations compared with measured data, with determination coefficients of 0.911 and 0.812 (multivariable regression [MLR] model, normalized difference VI [NDVI], soil adjusted VI [SAVI], enhanced VI [EVI], and difference VI [DVI]), 0.899 and 0.87 (quadratic polynomial regression, NDVI), and 0.749 and 0.829 (quadratic polynomial regression, NDVI) under low, medium, and high water levels, respectively. The LAI and SPAD derived from VIs had better potential in estimating winter wheat yield by using multivariable linear regressions, compared to the estimation yield based on VIs directly derived from UAV multispectral images alone by using linear regression, quadratic polynomial regression, and exponential models. When crop parameters (LAI and SPAD) in the flowering period were adopted to estimate yield by using multiple linear regressions, a high correlation of 0.807 was found, while the accuracy was over 87%. Importing LAI and SPAD obtained from UAV multispectral imagery based on VIs into the yield estimation model could significantly enhance the estimation performance. This study indicates that the multivariable linear regression could accurately estimate winter wheat LAI, SPAD, and yield under different water treatments, which has a certain reference value for the popularization and application of UAV remote sensing in precision agriculture.

## Introduction

The estimation of crop parameters (leaf area index [LAI] and chlorophyll content) is helpful in improving the level of crop monitoring, which is key to accurate monitoring and estimation of crop growth in agricultural management (Huang et al., 2016; Li et al., 2016; Liu et al., 2017; Yebra et al., 2017; Sun et al., 2021). LAI and chlorophyll are often used to describe canopy structure and to predict grain yield (Guo et al., 2020), which requires efficient and rapid measurement of crop LAI and soil and plant analyzer development (SPAD, which is used instead of chlorophyll content). Traditional methods to estimate crop parameter are based on destructive measurement, which not only consume time and manpower but also are difficult to be applied in a large area. In recent decades, remote sensing technology has been successfully applied to crop growth monitoring through satellite platforms, manned airborne platforms, and ground spectral equipment (Michele et al., 2015; Maimaitijiang et al., 2017; Ansar and Muhammad, 2020; Dehkordi et al., 2020). There are two kinds of satellite remote sensing data for crop parameters, namely, optical image and synthetic aperture radar data (Cougo et al., 2015; Castillo et al., 2017; Du et al., 2017; Pham and Yoshino, 2017; Pandit et al., 2018; Li et al., 2019), providing different spatial resolutions, such as SPOT (20 m), MODIS (250 m), Sentinel 1A (10 m), and ALOS-2 PLASAR2 (6 m) (Niu et al., 2019). However, several limitations such as deficient spatiotemporal resolution and cloud cover contamination restrain the application of satellite-based platforms. Relatively, the operation cost of manned airborne platforms is relatively high, and ground-based spectral devices are laborious and suffer from inefficient operations (Zhang and Kovacs, 2012; Yang et al., 2017; Yao et al., 2017; Katja et al., 2018).

In contrast, the rapid development of unmanned aerial vehicle (UAV) platforms provides an economical and efficient method to meet the increasing requirements of spatial, temporal, and spectral resolutions (Yue et al., 2017; Zheng et al., 2018; Heinemann et al., 2020; Qiao et al., 2020). UAV-based multispectral images were adopted to predict crop growth status and to predict grain yield in recent years. For example, Yao et al. (2017) obtained narrowband multispectral images based on UAV and used MTVI2 to estimate wheat LAI effectively, with an accuracy of 0.79 and a relative root mean square error (RMSE) of 24%. Guo et al. (2018) obtained remote sensing images based on UAV and established an inversion model of mangrove LAI by using the vegetation-level interruption index (VLOI), with an inversion accuracy of 0.72 and an RMSE of 0.137. Gao et al. (2016) used a multirotor UAV synchronously carrying a Canon Power Shot G16 digital camera and ADC Lite multispectral sensor to obtain the crown (Tian et al., 2016). Fu et al. (2020) examined the ability of multiple image features derived from UAV RGB images for winter wheat N status estimation across multiple critical growth stages. Another difference of the study on UAV-based prediction of plant LAI was that researchers usually aimed at a few growth periods (Hua et al., 2012). There is still little information on using UAVs to predict plant LAI during the whole important growth stages on a large scale of LAI, and fewer had studied the accuracy comparison of UAV inversion of LAI under different water treatments. The chlorophyll concentration, measured in mass per unit leaf area, is an important biophysical parameter retrievable from reflectance data (Zhu et al., 2020). Tian et al. (2016) used the spectral index of UAV imaging spectrometry to retrieve the chlorophyll concentration of cotton using multiple stepwise regression and partial least squares regression and achieved high accuracy. The Hekou District was selected as the core test area, and 140 ground sampling points were selected. Based on the measured SPAD values and UAV multispectral images, UAV-based SPAD inversion models were constructed, and the most accurate model was selected (Zhang et al., 2019). Cao J. et al. (2020) developed an inversion model that can predict japonica rice chlorophyll content by using the hyperspectral image of the rice canopy collected with a UAV. The inversion model was developed by using an extreme learning machine (ELM), the parameters of which are optimized by using particle swarm optimization (PSO). The PSO-ELM algorithm could accurately model the nonlinear relationship between hyperspectral data and chlorophyll content. The model achieved a coefficient of determination (*R*^2^) of 0.791 and an RMSE of 8.215 mg/L. Furthermore, UAVs are promising remote sensing platforms that is gaining more and more attention for crop studies. For example, Jin et al. (2017) estimated wheat plant density from UAV RGB images. Zhou et al. (2017) estimated grain yield in rice using multitemporal vegetation indices (VIs) from UAV-based multispectral and digital imagery. This parameter has been extensively studied in the field of remote sensing, while research for multispectral data based on UAV is relatively few under different water treatments. The techniques used are mainly based on portable spectrometers, airborne multispectral imagers, and remote sensing satellites (Skudra and Ruza, 2017). Portable spectrometers have difficulty differentiating “point” from “surface,” while satellite images have coarser spatial resolution and poor timeliness and are thus prone to “isospectral foreign bodies,” resulting in low prediction accuracy. Therefore, the prediction of chlorophyll concentrations and LAI using multispectral sensors on low-altitude UAV remote sensing platforms has gradually become a trend (Cao Y. et al., 2020; Guo et al., 2020; Wan et al., 2020). The existing researchers used the multispectral remote sensing images of medium and low spatial resolutions (such as Landsat 8 and TM) to carry out remote sensing inversion research on winter wheat LAI, SPAD, yield estimation, and other indicators. However, due to the limitations of spatial resolution, revisit period, weather conditions, and other factors, there are still some limitations in the precise monitoring of winter wheat growth.

Therefore, this study is aimed at estimating LAI, SPAD, and yield of winter wheat based on VIs (normalized difference VI [NDVI], soil adjusted VI [SAVI], enhanced VI [EVI], and difference VI [DVI]) derived from UAV multispectral images. The estimation performances of models based on VI alone and VI combinations were also analyzed. According to the obtained optimal estimation of LAI and SPAD values, the crop yield was estimated. More specifically, our study paid attention to the following:

(1) Establishment of winter wheat LAI and SPAD estimation models under different water treatments based on VI alone by using a linear regression model, quadratic polynomial regression model, and exponential regression analysis and based on VI combinations by multivariable regression (MLR) analysis;
(2) A comparison of the performances of winter wheat LAI and SPAD estimating models and selection of the optimal estimation models of LAI and SPAD;
(3) Estimation of the winter wheat yield by using multivariate regression model based on the optimal LAI and SPAD values obtained in (2) in the flowering stage.

## Materials and Methods

### Study Area

**FIGURE 1:**
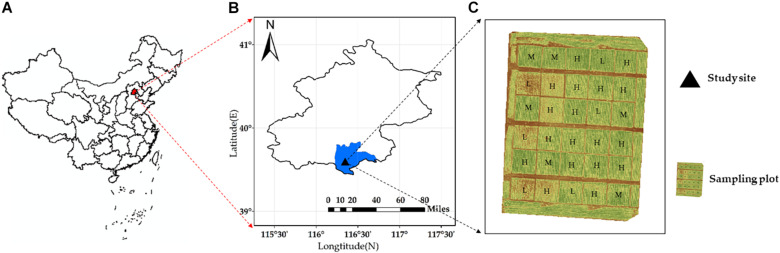
Research area: **(A)** location of the research field in China; **(B)** area view of the research field indicating region division; and **(C)** the location of sampling plots and ground control plots; L, M, and H represent low water, medium water, and high water treatments, respectively.

**FIGURE 2:**
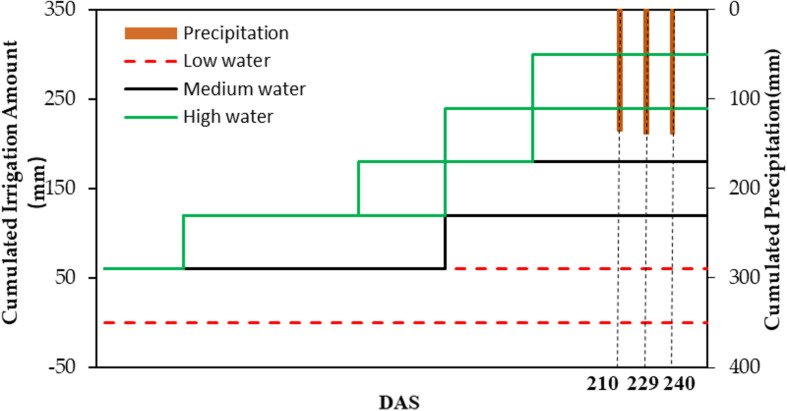
Irrigation and precipitation accumulation under different irrigation treatments (low water, medium water, and high water).

The experiment was carried out on a field in the Daxing District located in the south of Beijing, China (39°37.25′N, 116°25.51′E). The research field with an area of approximately 1.68 km^2^ was planted with winter wheat. Thirty 7.8 × 7.5-m^2^ fields within each region were chosen as samples for data collection (Figure 1). According to the amount of irrigation, the 30 fields were divided into three irrigation levels (low water [0–60 mm], medium water [120–180 mm], and high water [240–300 mm]). The cumulated precipitation rates of DAS210 (days after sowing [DAS], jointing stage), DAS229 (flowering stage), and DAS240 (filling stage) were 134.87, 138.43, and 138.43 mm, respectively (Figure 2). The soil type was loam sandy (85% sand, 11.5% power, and 3.5% clay), according to the United States Department of Agriculture taxonomy. Winter wheat was planted on October 5, 2017, and harvested on June 30 with a 265-day life span (Table 1).

**TABLE 1:** Crop management 2017–2018.

Item	Winter wheat
Sowing date	October 5, 2017
Variety	Zhong Mai 175
Average seeding rate	289 grains/m^2^
Harvest date	June 30, 2018
Soil type	Loam sandy

### Research Measurements

At the winter wheat key stages (DAS210–DAS240) (Du et al., 2017; Yue et al., 2017; Ji et al., 2020; Jiang et al., 2020; Tao et al., 2020a), crop parameters (LAI and SPAD) were measured on DAS210, DAS229, and DAS240. Sixty sets of samples (LAI [30 sets] and SPAD [30 sets]) were obtained every 7 days.

LAI from LAI-2200C: To be synchronous with the imagery, we took three wheat stems from each plot as one sample, separated the green leaves, and used an LI-3000 leaf area meter to scan the green leaf area. We had a total of 18 plots (low water: six plots; medium water: six plots; and high water: six plots), whose length and width are 7.8 and 7.5 m, respectively. The number of stems 1 m in length in each plot was counted manually. Based on formula (1), the LAI of the population was calculated.

(1) $$LAI=\frac{1}{D}\times B\times \frac{A}{C}\times 10^{-4}$$

where *D* is the distance between two rows of wheat; *B* is the number of stems in 1 m in length; *A* is the leaf area of the sample; and *C* is the number of stems of the sample. A SPAD-502 chlorophyll meter (Minolta Corporation, NJ, United States) was used for *in vivo* measurement of the ratio of light transmittance through the leaf. Instrument readings have been shown to correlate well with laboratory measurements of chlorophyll concentrations in several species (Haie and Keller, 2008). On each sampling campaign, 30 SPAD measurements were collected on average. The chlorophyll meter readings were taken midway on fully expanded top-of-canopy leaves. Each measurement was characterized by the mean of three replicate measurements. The chlorophyll meter measured an area of 2 × 3 mm with an accuracy of ± 1.0 SPAD unit (at room temperature).

### Acquisition and Pretreatment of UAV Multispectral Image

In this study, a small six-spin UAV (Nanjing Hepu Aero Science and Technology Co., Ltd.)^1^ was used. Multispectral cameras were mounted synchronously on a UAV remote sensing platform (before the camera was used, noise removal and lens distortion correction were carried out). Table 2 illustrates the UAV specification in more detail.

**TABLE 2:** UAV and multispectral camera specifications.

UAV		Multispectral camera	
Type	Pixhawk (M600)	Type	Red Edge-M
Maximum payload	5 kg	Band range	475–840 nm
Maximum duration	15 min	Terrestrial resolution	0.0409 m

When the weather was clear and cloudless, three flights (DAS 210, DAS229, and DAS240) were carried out from 12:00 to 13:00, when the solar zenith angle was minimal. Continuous flight monitoring was carried out in 30 plots of the study area. The flight altitude was 60 m, and the spatial resolution was 0.0409 cm.

The image mosaic processing was performed by using the Pix4Dmapper software (Pix4D Inc., Switzerland)^2^ (Turner et al., 2012). The preprocessing of mosaic multispectral images included geometric correction and radiation correction, and the geometric correction mainly used the ENVI software. With the Orthophoto image as a reference image, 20 reference points were selected uniformly in different positions of the image to correct the geometric accuracy of the multispectral image. The error of the geometric correction of the image was less than 0.5 pixels after verification. For radiation correction, due to the difference between the time and weather conditions of the multispectral data obtained from different sites, the pseudo-standard ground object radiation correction method was used to convert the multispectral image value into the image reflectance value through the reflectance measured by the ground target (Wang and Liu, 2014).

**FIGURE 3:**
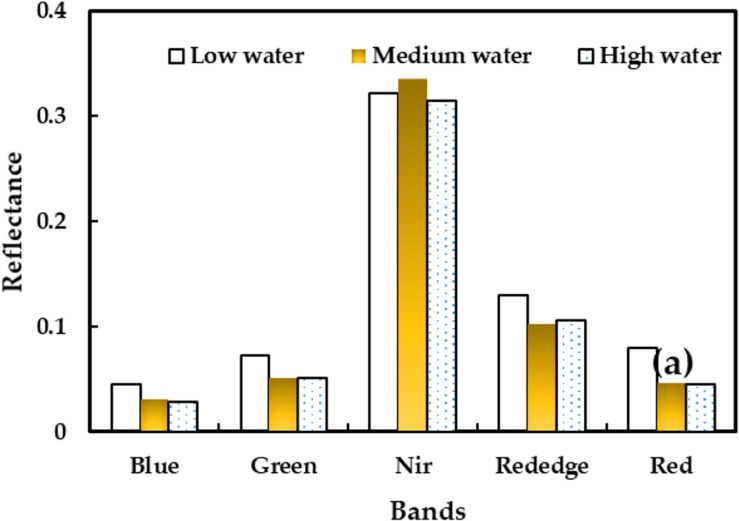
Reflectance of five bands under different water treatments.

The five multispectral bands were blue (central wavelength 475 nm, bandwidth 40 nm), green (central wavelength 560 nm, bandwidth 40 nm), red (central wavelength 668 nm, bandwidth 40 nm), red edge (central wavelength 717 nm, bandwidth 10 nm), and near infrared (central wavelength 840 nm, bandwidth 40 nm) (Figure 3).

### UAV Multispectral VI

**FIGURE 4:**
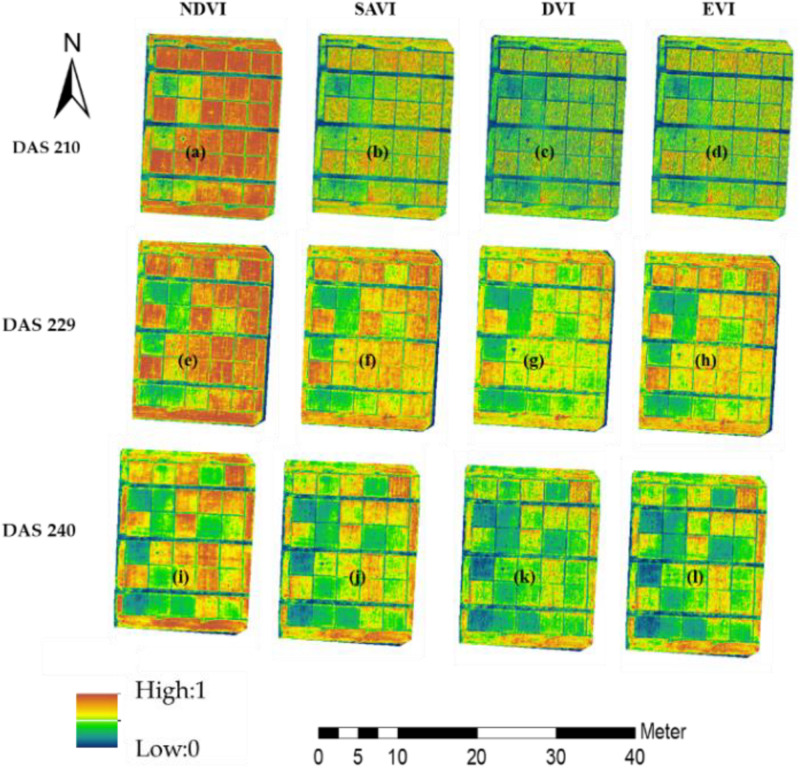
VI of the research area. **(a–d)** were the NDVI, SAVI, SVI and EVI in DAS210 of research area, **(e–h)** were the NDVI, SAVI, SVI and EVI in DAS229 of research area, **(i–l)** were the NDVI, SAVI, SVI and EVI in DAS240 of research area.

Many previous studies have used different VIs in multispectral imagery to estimate the crop parameters (LAI and SPAD). In this study, VIs and one VI combination were calculated by using visible bands, including the NDVI (Rouse et al., 1974), SAVI (Huete, 1988), EVI (Huete et al., 2002), and DVI (Jordan, 1969; Figure 4). Their calculation formulas are as follows:

(2) $$NDVI=\frac{R_{nir}-R_{red}}{R_{nir}+R_{red}}$$

(3) $$SAVI=1.5\frac{R_{nir}-R_{red}}{R_{nir}+R_{red}+0.5}$$

(4) $$EVI=2.5\frac{R_{nir}-R_{red}}{R_{nir}+6R_{red}-7.5R_{blue}+1}$$

(5) $$DVI=R_{nir}-R_{red}$$

Note that *R*_*nir*_ is the near-infrared reflectance, *R*_*red*_ is the red reflectance, and *R*_*blue*_ is the blue reflectance.

### Estimation Models of Crop Parameters (LAI and SPAD) and Yield

**FIGURE 5:**
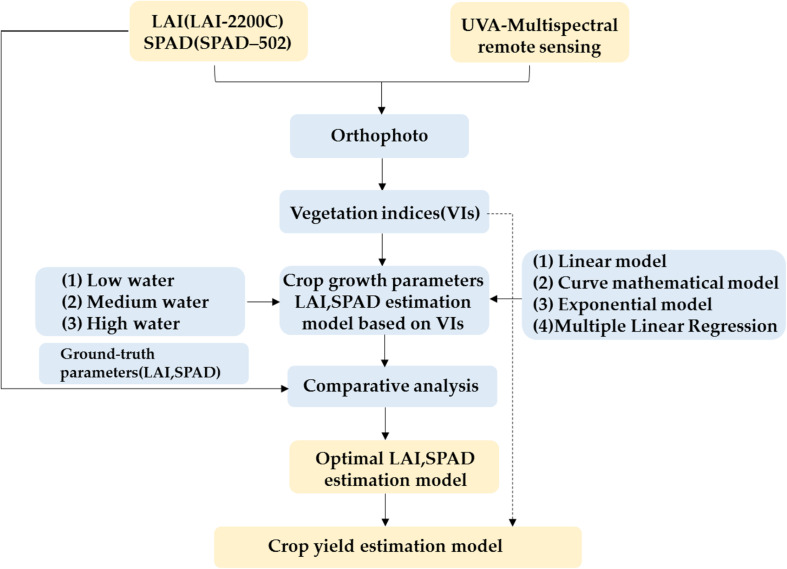
Schematic indicating the main procedures to obtain the optimal estimation model of winter wheat crop parameters and yield.

Figure 5 showed the main procedures of obtaining the optimal estimation model of crop parameters and yield based on winter wheat features derived from UAV multispectral imagery. Four estimation models of winter wheat crop parameters were used in this study, i.e., prediction models: (1) linear regression model, (2) quadratic polynomial regression, (3) exponential model, and (4) multiple linear regression based on VIs. In the establishment of the yield prediction model, multivariable linear regression analysis was adopted.

### Statistical Analysis

For statistical analysis, SPSS 23 was adopted. For the spectral reflectance information of winter wheat observed in different plots, the linear regression model, quadratic polynomial regression, exponential model, and multiple linear regression model (Quan et al., 2017) of winter wheat VIs, LAI, and SPAD were established. The validation set was used to fit the predicted and measured values of the model (Marenco et al., 2009; Drusch et al., 2012), and then the multiple linear regression model was used to predict the output.

(6) y=ax+b

(7) $$\text{y}=\text{a}x^{2}+\text{bx}+c\;$$

(8) $$\text{y}=\text{a}e^{x}$$

(9) $$\text{y}=y_{0}+y_{1}x_{1}+y_{2}x_{2}+\cdots +y_{n}x_{n}+m$$

In the formula, *y*_0_, *y*_1_, *y*_2_, …, *y*_*n*_ is the regression coefficient, and *m* is the model error.

The coefficient of determination (*R*^2^) and RMSE were used to evaluate the performance of each model. Mathematically, a higher *R*^2^ corresponds to a smaller RMSE and thus represents better model accuracy. The following equations were used to calculate *R*^2^ and RMSE (Pandit et al., 2018; Li et al., 2019), respectively:

(10) $$R^{2}=\frac{\sum_{\text{i}=1}^{\text{n}}{\left(y_{i}-x_{i}\right)}^{2}}{\sum_{\text{i}=1}^{\text{n}}{\left({\text{y}}_{\text{i}}-\bar{\text{y}}\right)}^{2}}$$

(11) $$\text{RMSE}=\sqrt{\frac{\sum_{\text{i}=1}^{\text{n}}{\left(x_{i}-{\text{y}}_{\text{i}}\right)}^{2}}{\text{n}}}$$

where *x*_*i*_ and *y*_*i*_ are the estimated and measured values, respectively; $\bar{x}$ and $\bar{y}$ are the average estimated and measured values, respectively; and *n* is the sample number.

## Results

Table 3 shows the statistics of LAI, SPAD, and yield measurements for different water treatments. In this study, four VIs (NDVI, SAVI, EVI, and DVI) derived from UAV multispectral imagery were used for the linear regression model, quadratic polynomial regression, exponential model, and multiple linear regression for winter wheat LAI and SPAD under low water, medium water, and high water. In Figures A1–A3 and Tables 4, 5, the four VIs all had significant positive correlations (*p* < 0.01) with low water, medium water, and high water on LAI and SPAD.

**TABLE 3:** Descriptive statistics of LAI, SPAD, and yield from the study area.

Treatment	Parameter	Samples	Min	Mean	Max	SD	CV (%)
Low water	LAI	18	2.00	3.68	5.04	0.96	25.98
	SPAD	18	42.08	51.78	63.97	6.37	12.30
	Yield (kg/ha)	6	2,397.0	4,945.4	3,731.2	944.91	25.12
Medium water	LAI	18	2.53	4.22	5.67	0.97	22.93
	SPAD	18	42.36	53.44	63.08	6.97	13.03
	Yield (kg/ha)	6	2,935.3	5,588.6	4,526.4	1,093.4	24.23
High water	LAI	18	2.97	3.97	5.99	0.65	11.35
	SPAD	18	50.66	60.85	72.67	6.91	16.39
	Yield (kg/ha)	6	3,116.3	4,848.6	5,810.7	926.1	19.02

*SD, standard deviation; CV, coefficient of variation. Six points were randomly selected from 18 points of high water treatment.*

**TABLE 4:** Correlations between VIs derived from UAV multispectral imagery and LAI with different water treatments.

Model	VIs	Low water		Medium water		High water	
		*R*^2^	RMSE	*R*^2^	RMSE	*R*^2^	RMSE
Linear	NDVI	0.8504**	0.606	0.8943**	0.6222	0.6673**	0.5992
	SAVI	0.6972**	0.5979	0.818**	0.6095	0.7395**	0.6317
	EVI	0.7455**	0.624	0.7643**	0.5924	0.7467**	0.6345
	DVI	0.6609**	0.5915	0.7586**	0.5995	0.7162**	0.6338
Quadratic polynomial	NDVI	0.8676**	0.5914	0.8951**	0.6223	0.7493**	0.6312
	SAVI	0.6973**	0.5979	0.8523**	0.6189	0.7409**	0.6317
	EVI	0.7466**	0.6065	0.8746**	0.6152	0.7261**	0.6298
	DVI	0.6637**	0.6272	0.8208**	0.6116	0.7461**	0.6235
Exponential	NDVI	0.7961**	0.6583	0.8966**	0.6094	0.6504**	0.6081
	SAVI	0.6983**	0.6299	0.8223**	0.6357	0.7249**	0.6551
	EVI	0.6467**	0.6178	0.7241**	0.6112	0.73**	0.6592
	DVI	0.6197**	0.8136	0.7175**	0.6258	0.7038**	0.6610
Multiple	Four VIs	0.911**	0.2251	0.73**	0.6558	0.744**	0.637

Four VIs, NDVI, SAVI, EVI, and DVI, were used to establish regression estimation models for LAI with different water treatments. **Significant at the level of 0.01.

**TABLE 5:** Correlations between VIs derived from UAV multispectral images and SPAD with different water treatments.

Model	VIs	Low water		Medium water		High water	
		*R*^2^	RMSE	*R*^2^	RMSE	*R*^2^	RMSE
Linear	NDVI	0.7994**	7.205	0.8693**	7.632	0.748**	6.3045
	SAVI	0.7423**	7.089	0.8249**	7.541	0.7061**	6.5237
	EVI	0.7222**	7.048	0.7011**	7.281	0.7112**	6.5348
	DVI	0.6831**	7.968	0.7842**	7.456	0.6549**	6.4787
Quadratic polynomial	NDVI	0.8082**	7.022	0.8697**	7.633	0.8296**	6.1259
	SAVI	0.7689**	7.143	0.8306**	7.553	0.7247**	6.6320
	EVI	0.7485**	7.102	0.803**	7.514	0.7217**	6.6398
	DVI	0.7375**	7.079	0.7491**	7.591	0.6637**	6.5622
Exponential	NDVI	0.7788**	7.341	0.8742**	7.038	0.7369**	6.4004
	SAVI	0.7212**	7.172	0.8243**	8.951	0.6789**	6.6773
	EVI	0.6989**	7.121	0.7034**	7.4651	0.6813**	6.6899
	DVI	0.6627**	7.989	0.7812**	7.599	0.6225**	6.6248
Multiple	Four VIs	0.812**	7.018	0.87**	7.059	0.8221**	6.412

Four VIs and one VI combination, NDVI, SAVI, EVI, and DVI, were used to establish regression estimation models for SPAD with different water treatments. **Significant at the level of 0.01.

### Estimation Models of Winter Wheat LAI on the Basis of UAV Multispectral VIs

As shown in Table 4, the NDVI had the highest *R*^2^ values of 0.868 (quadratic polynomial), 0.897 (exponential), and 0.749 (quadratic polynomial), followed closely by the EVI with *R*^2^ of 0.747 (quadratic polynomial), 0.8746 (quadratic polynomial), and 0.741 (quadratic polynomial) and by the and SAVI with *R*^2^ of 0.698 (exponential), 0.852 (quadratic polynomial), and 0.740 (linear). The lowest correlations were observed by using DVI to estimate winter wheat, with *R*^2^ values of 0.620 (exponential), 0.718 (exponential), and 0.607 (exponential). When it comes to the RMSE, a similar observation was obtained. And for low-water LAI, the NDVI also had a lower RMSE of 0.591, followed closely by the EVI and SAVI with RMSE values of 0.607 and 0.615, respectively. The DVI had a larger RMSE of 0.814. For medium and high water treatments, similar observations were found.

**FIGURE 6:**
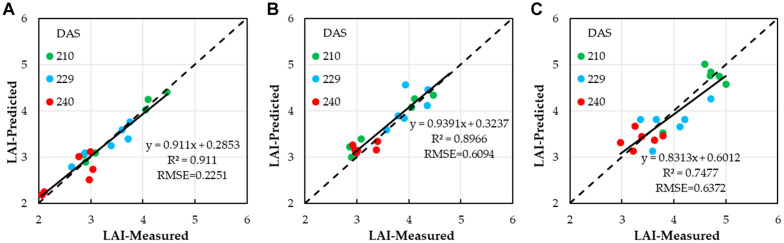
Correlations between winter wheat LAI estimated based on NDVI, SAVI, EVI, and DVI by using optimal regression analysis and ground-truth LAI; **(A)** lower water treatment, **(B)** medium water treatment; **(C)** and higher water treatment.

After estimating winter wheat LAI with different water treatments by using a single VI, for LAI with low water treatment (*LAI*_*l*_), the four VI combinations that had the higher correlations were chosen to estimate winter wheat LAI by adopting MLR analysis. When MLR was used, the estimation performance of winter wheat LAI was improved (Figure 6A), with an *R*^2^ value of 0.911, with an increase of 0.0434 compared to the highest *R*^2^ value of 0.8676 (NDVI, quadratic polynomial) for the single VI. The RMSE of LAI decreased to 0.3663, compared to the lowest RMSE value of 0.5914 for single VI, quadratic polynomial. The winter wheat LAI could be calculated based on NDVI, SAVI, EVI, and DVI by using Equation (12). However, for LAI with medium and high water treatments (*LAI*_*m*_ and *LAI*_*h*_), when MLR was adopted, the estimation performance of winter wheat (*LAI*_*m*_ and *LAI*_*h*_) was not improved, and the *R*^2^ values were 0.73 and 0.744, with a decrease of 0.167 and 0.005, compared to the highest *R*^2^ values of 0.897 (NDVI, exponential) and 0.748 (NDVI, quadratic polynomial) for the single VI. The RMSE of LAI increased by 0.046 and 0.058, compared to the lowest RMSE values of 0.609 and 0.631 for single VI with single linear regression, quadratic polynomial, and exponential models, respectively. The winter wheat (*LAI*_*m*_ and *LAI*_*h*_) could be calculated based on NDVI by using Equations (13) and (14) (Figures 6B,C).

(12) $$\begin{array}{c} LAI_{l}=14.643\times NDVI-15.293\times SAVI+33.510\\ \times EVI-16.431\times DVI-0.698 \end{array}$$

(13) $$LAI_{m}=1.3626\times e^{1.384NDVI}$$

(14) $$LAI_{h}=13.567\times NDVI^{2}-14.567\times \text{NDVI}+6.932$$

### Prediction Models of Winter Wheat SPAD Based on UAV Multispectral VIs

In Table 5, the NDVI had the highest correlations with *R*^2^ values of 0.808 (quadratic polynomial), 0.874 (exponential), and 0.829 (quadratic polynomial) followed by the SAVI with *R*^2^ values of 0.769 (quadratic polynomial), 0.831 (quadratic polynomial), and 0.725 (quadratic polynomial). For both low and high water treatments, the lowest correlations were observed by using DVI to predict SPAD, with *R*^2^ values of 0.663 (exponential) and 0.623 (exponential), respectively. However, with the medium water treatment, the lowest correlations were observed by using EVI to estimate SPAD, with *R*^2^ values of 0.701 (linear). For low water level, the NDVI also had the lowest RMSE with a value of 7.00, followed by the SAVI with an RMSE value of 7.17; the maximum RMSE of DVI was 7.99. For the medium and high water treatments, similar observations were found. The *R*^2^ was negatively correlated with RMSE.

**FIGURE 7:**
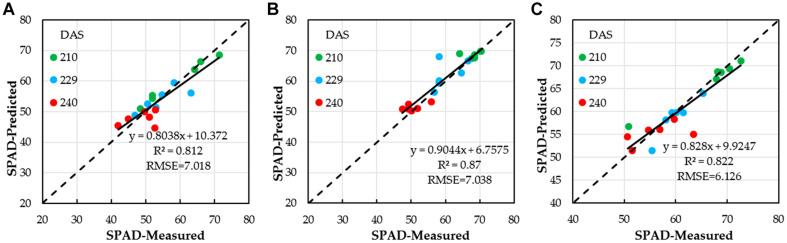
Correlations between winter wheat SPAD estimated based on NDVI, SAVI, EVI, and DVI by using optimal regression analysis and ground-truth SPAD; **(A)** low water treatment, **(B)** medium water treatment; and **(C)** high water treatment.

After estimating winter wheat SPAD with different water treatments by using single VI, for SPAD with low water treatment (*SPAD*_*l*_), the four VI combinations, which had the highest correlations, were chosen to estimate winter wheat SPAD by using MLR analysis. When MLR was used, the estimation performance of winter wheat *SPAD*_*l*_ was improved (Figure 7A); the *R*^2^ value was 0.812, with an increase of 0.0038, compared to the highest *R*^2^ value of 0.808 (NDVI, quadratic polynomial) for the single VI. The RMSE of SPAD decreased with a value of 0.004, compared to the lowest RMSE value of 7.022 for single VI, quadratic polynomial. The winter wheat *SPAD*_*l*_ could be calculated based on NDVI, SAVI, EVI, and DVI by using Equation (15). However, with medium and high water treatments (*SPAD*_*m*_ and *SPAD*_*h*_), when MLR was used, the estimation of winter wheat (*SPAD*_*m*_ and *SPAD*_*h*_) was not improved, and the *R*^2^ values were 0.87 and 0.822, with a decrease of 0.0042 and 0.008, respectively, compared to the highest *R*^2^ values of 0.8742 (NDVI, exponential) and 0.830 (NDVI, quadratic polynomial) for the single VI. The RMSE of SPAD increased with values of 0.021 and 0.286, compared to the RMSE of 7.038 and 6.126 for single VI with the single linear regression, quadratic polynomial, exponential models. The winter wheat (*SPAD*_*m*_ and *SPAD*_*h*_) could be calculated based on NDVI by using Equations (16) and (17) (Figures 7B,C).

(15) $$\begin{array}{c} SPAD_{l}=249.19\times NDVI-686.477\times SAVI+24.896\\ \times EVI+596.61\times DVI+24.647 \end{array}$$

(16) $$SPAD_{m}=26.499\times e^{1.0796\times NDVI}$$

(17) $$SPAD_{h}=108.21\times NDVI^{2}-103.02\times NDVI+74.93$$

### Prediction Models of Winter Wheat Yield on the Basis of Both LAI and SPAD

**FIGURE 8:**
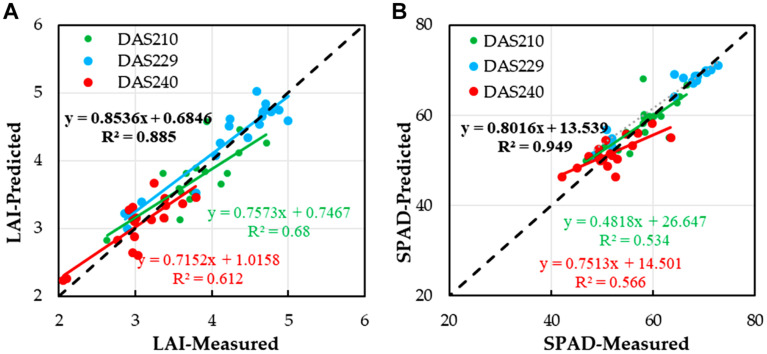
Relationships between LAI, SPAD, and yield of winter wheat under different stages; **(A)** LAI and **(B)** SPAD.

Remote sensing estimation of winter wheat yield is based on VIs which can reflect crop yield. It is necessary to verify whether the relationship between wheat LAI, SPAD, and yield (measured) is significant. Figure 8 shows the relationship between LAI, SPAD, and winter wheat yield under different stages. It can be seen that the relationships between LAI, SPAD, and winter wheat yield were the most significant in different models. LAI and SPAD could estimate winter wheat yield well under different stages. For LAI (Figure 8A) and SPAD (Figure 8B), *R*^2^ values are 0.68 (DAS210), 0.885 (DAS229), and 0.612 (DAS240) and 0.534 (DAS210), 0.949 (DAS229),and 0.566 (DAS240), respectively. This provided a basis for the prediction of winter wheat yield using LAI and SPAD which are estimated by VIs.

Figures 8A,B show that the predicted values of crop growth parameters at three stages (DAS210, DAS229, and DAS240) were consistent with the measured values and that the R^2^ values (0.885 for LAI and 0.949 for SPAD) were the highest in DAS229, which was the best estimation period of yield; this result was consistent with that of previous studies (Zhang et al., 2019; Tao et al., 2020b). Under the support of the above, the optimal estimates of winter wheat LAI and SPAD values in DAS229 based on VIs were adopted to estimate the yield of winter wheat by using the quadratic polynomial model (Equations (18) and (19) and Figure 10). The accuracy of LAI and SPAD based on UAV multispectral imagery to estimate winter wheat yield was over 87%.

**FIGURE 9:**
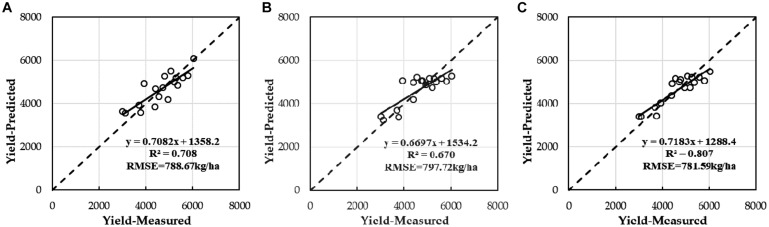
Correlations between yield of winter wheat estimated based on LAI and/or SPAD alone by using optimal regression analysis and multivariable liner regression with ground-truth yield; **(A)** LAI, **(B)** SPAD, and **(C)** LAI and SPAD.

**FIGURE 10:**
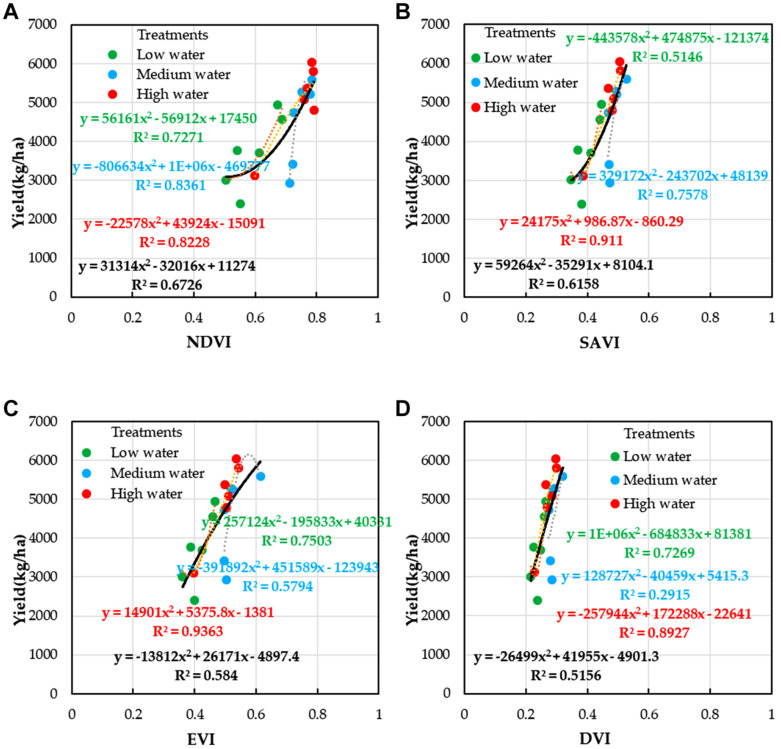
Relationships between VIs and yield of winter wheat under different water treatments; **(A)** NDVI, **(B)** SAVI, **(C)** EVI, and **(D)** DVI.

(18) $$\text{Yield}=1095\times LAI^{2}-7666.6LAI+16981$$

(19) $$\text{Yield}=-2.214\times SPAD^{2}-366.9SPAD-9689.2$$

(20) Yield=327.44×LAI+72.15SPAD-1414.8

After estimating winter wheat yield based on a single parameter (LAI or SPAD) by using quadratic polynomial, when MLR was used in Equation (20), the estimation result of the winter wheat yield was improved (Figure 10C), and the *R*^2^ value was 0.807, with the increase of 0.099 and 0.137, respectively, compared to the *R*^2^ values of 0.708 (LAI) and 0.670 (SPAD) for the single parameter. The RMSE of yield decreased by 7.08 and 16.13 kg/ha, compared to the RMSE values of 788.67 kg/ha (LAI, quadratic polynomial) and 797.72 kg/ha (SPAD, quadratic polynomial) for the single parameter, respectively.

## Discussion

### Estimation of Winter Wheat Parameters at Different Water Treatments

The estimation of winter wheat LAI and SPAD with different water treatments (low water, medium water, and high water) using linear, quadratic polynomial, exponential models in different days is shown in Appendix Figures A1–A3. It is shown that the best agreement of predicted winter wheat LAI and SPAD values was for the medium water level (120–180 mm), followed by the low water level (0–60 mm) (LAI); the worst was the high water level (210–240 mm). In Table 4, the *R*^2^ values between the LAI obtained from eight estimation models and the measured LAI were more than 0.7 in the medium water level (0.7175–0.8966, at low water level: 0.6197–0.8504 and at high water level: 0.650–0.749); there were few estimation models with *R*^2^ greater than 0.8 between the estimated LAI and the measured LAI. This indicates that different water treatments had an effect on the inversion of LAI-based VIs alone under the same models. However, when multivariable liner regression was adopted (Table 4 and Figure 6), the trend of LAI retrieval from VI-based UAV multispectral imagery had changed (Quan et al., 2017; Berger et al., 2018; Guo et al., 2020). The best inversion result was for the low water level, followed by the medium water level and high water level.

Again, it can be seen from Table 5 that all the *R*^2^ values between the SPAD obtained estimation models and the measured SPAD were more than 0.7 in medium water level (0.7011–0.8742); in both low water and high water levels, 75% of them have an *R*^2^ greater than 0.7, but in general, the estimation in the low water level was better. However, when the multivariable linear regression was adopted (Table 5 and Figure 7), the results here were not similar to the LAI; the best inversion result was for the medium water level, followed by the high water level and low water level. It is shown that it was necessary to consider multiple VIs to retrieve LAI and SPAD using multivariable linear regression compared to the VI alone (Sun et al., 2012; Boegh et al., 2013; Elarab et al., 2015; Li et al., 2015).

### Prediction of Winter Wheat Yield on the Basis of UAV

The crop parameters of winter wheat under different stages could reflect the change of yield (Lin et al., 2009; Sid”ko et al., 2017) (Figures 8A,B); the nonlinear values were both very significant (*R*^2^ = 0.885 for LAI and *R*^2^ = 0.949 for SPAD), indicating that the yield could be estimated by measuring LAI and SPAD of winter wheat in the flowering stage (DAS229). The LAI and SPAD of winter wheat were estimated by VI constructed by UAV multispectral imagery (NDVI, EVI, SAVI, and DVI) (Figures A1–A3). Figure 10 shows the estimation of winter wheat yield with NDVI, SAVI, EVI, and DVI constructed by UAV multispectral imagery under different water treatments, and NDVI had the highest correlation with an *R*^2^ value of 0.673 (medium water > high water > low water), followed by SAVI with an *R*^2^ value of 0.616 (high water > medium water > low water).

According to the principle that LAI and SPAD were closely related to the yield (Simonetta et al., 2009; Fang et al., 2010; Bendig et al., 2013), it was feasible to carry out large-area remote sensing yield estimation based on various VIs constructed by UAV multispectral imagery (Figures 8–10). If drought caused wilting of winter wheat, withering of lower leaves, or excessive irrigation, crop development was abnormal, which could be reflected by the dynamic change of crop parameters retrieved by UAV multispectral remote sensing.

## Future Work

There is an increasing need for further raising awareness on the issue of improving estimation performance by using drone multispectral images and constructing crop vision systems to estimate crop parameters and yields. In order to reduce the error in the process of image acquisition and processing, there are still some problems to be solved. First of all, since the pixel value of the image is the reflectivity of the incoming sunlight, the variation of light conditions may lead to the variation of the image-derived features. To further explore the relationship between light changes and camera response (reflection) during UAV flight missions, better integration of light dynamics (UAV attitude, sun position, light scattering, clouds, etc.) is needed to describe light changes. The number and date of data points selected are important issues. In this study, different water treatments (lower water, medium water, and higher water) were selected for the dates from jointing stage to filling stage (DAS210, DAS229, and DAS240). In addition, further research is needed to validate these results for different crops and different sites.

## Conclusion

The UAV multispectral remote sensing system, as an important farmland-scale data acquisition tool, has great application potential in rapidly, accurately, and economically estimating farmland crop parameters and yields. The results confirmed that the visible light directly derived from UAV multispectral imagery had a high correlation with the measured LAI and SPAD. Compared to the linear regression model, the quadratic polynomial model, and the exponential model based on VIs alone, the MLR based on NDVI, SAVI, DVI, and EVI had higher correlations for both LAI and SPAD under low water treatment with *R*^2^ values of 0.911 and 0.812, respectively. The quadratic polynomial model based on NDVI alone had higher correlations for both LAI and SPAD under medium water treatment, with *R*^2^ values of 0.8996 and 0.87, respectively. However, under high water treatment, the exponential model performance was better than that of the linear model and quadratic polynomial model, with *R*^2^ values of 0.829 and 0.749, respectively. Under different water treatments, the optimal regression model was different, and with medium water treatment, the estimation was better for both LAI and SPAD. The relationships between the measured crop parameters and the measured yield were verified, and good results were obtained (*R*^2^ = 0.689 for LAI and *R*^2^ = 0.717 for SPAD).

The LAI and SPAD derived from VIs had better potential to estimate winter wheat yield; in the flowering stage, the *R*^2^ values of winter wheat yield estimation based on LAI (quadratic polynomial) and SPAD (quadratic polynomial) were 0.708 and 0.670, respectively. When MLR was used to estimate the yield based on LAI and SPAD, the result of winter wheat yield estimation was improved (*R*^2^ = 0.807, RMSE = 781.59 kg/ha). The ability of VI to identify different aspects of plants is different, which results in improving the prediction performance. Adding LAI and SPAD of UAV multispectral images into the production prediction model based on VIs can significantly improve the performance of production estimation.

In conclusion, this study shows the potential of the UAV multispectral imagery and regression model to estimate the growth parameters and yield of winter wheat. The results provide reference and technical support for the popularization and application of UAV remote sensing in large-scale precision agriculture.

## Data Availability Statement

The original contributions presented in the study are included in the article/supplementary material, further inquiries can be directed to the corresponding author/s.

## Author Contributions

XH, ZW, and BZ: conceptualization. XH: data curation. YL and ZW: funding acquisition. XH, HC, and ZW: methodology. YL: project administration. TD: supervision. XH: writing—original draft. HC and BZ: writing—review and editing. All authors contributed to the article and approved the submitted version.

## Conflict of Interest

The authors declare that the research was conducted in the absence of any commercial or financial relationships that could be construed as a potential conflict of interest.

## Footnotes

1 http://www.agrouas.com/

2 http://www.pix4d.com
