# Supplementary material for: Inversion of Winter Wheat Growth Parameters and Yield Under Different Water Treatments Based on UAV Multispectral Remote Sensing
Source: Front Plant Sci. 2021 May 20;12:609876. doi: 10.3389/fpls.2021.609876 (PMC8173193; doi:10.3389/fpls.2021.609876)
Supplement: Supplementary file 1 [file Data_Sheet_1.pdf]

APPENDIX

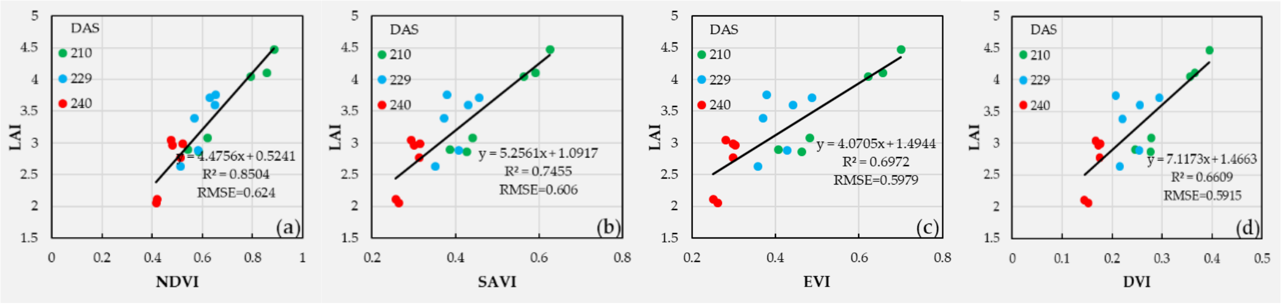

（i）

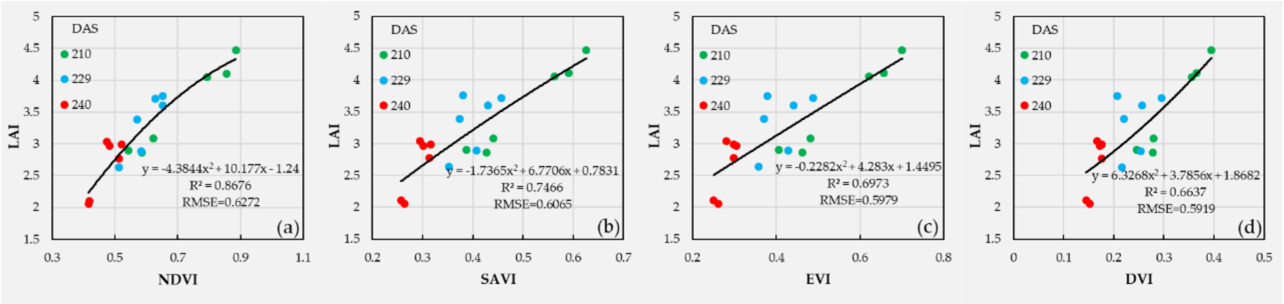

(ii)

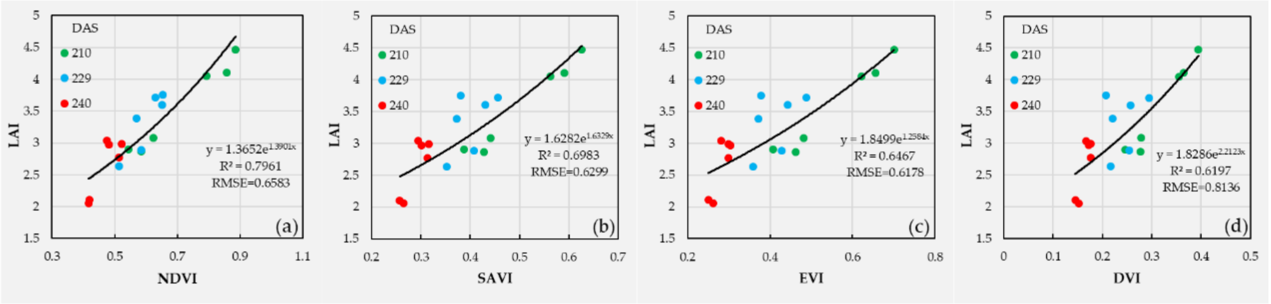

(iii)

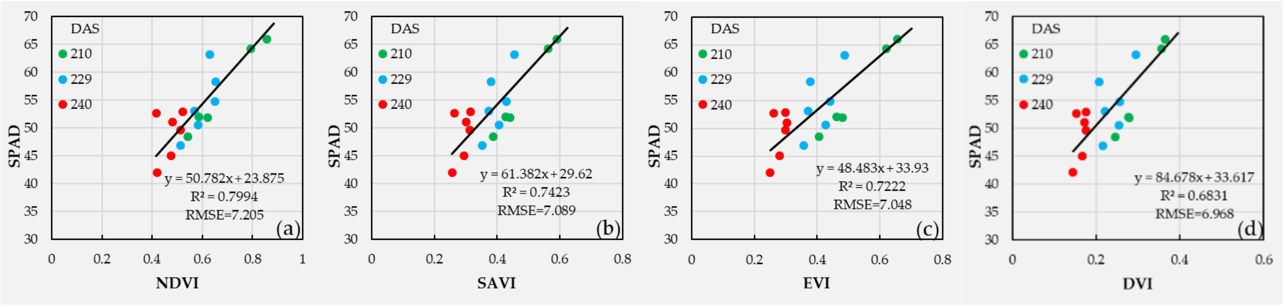

(iv)

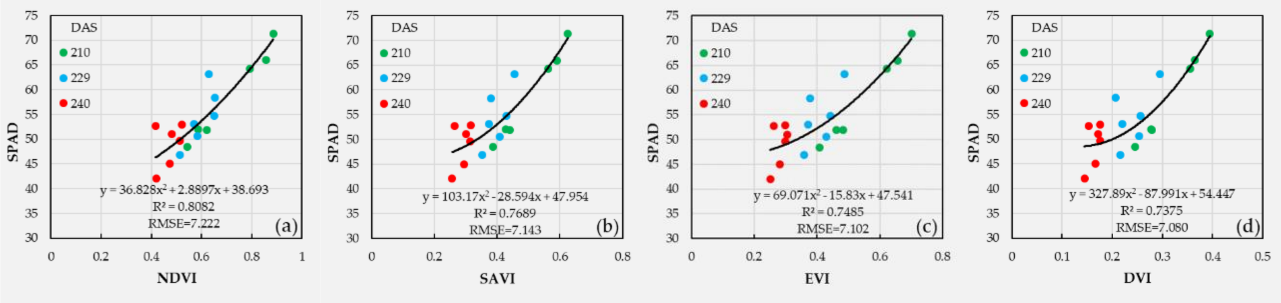

(v)

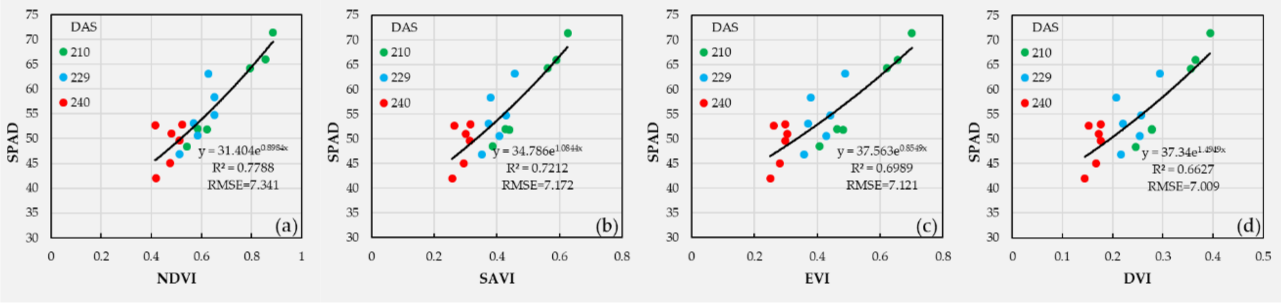

(vi)

Figure A1. LAI and SPAD estimation under Low water treatment based on VIs; (i) and (iv) were Linear regression model, (ii) and (v) were quadratic polynomial regression, (iii) and (vi) were exponential model.

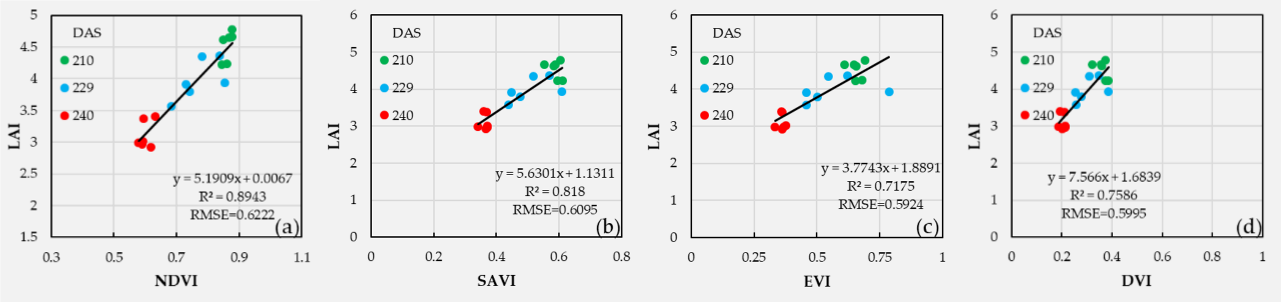

（i）

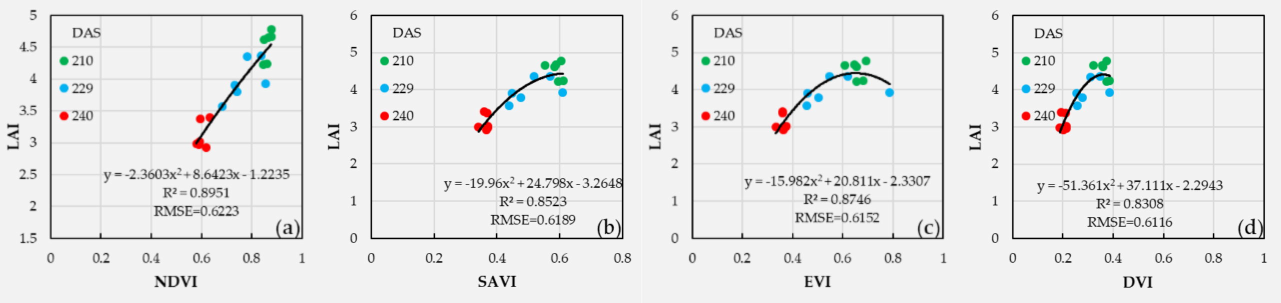

(ii)

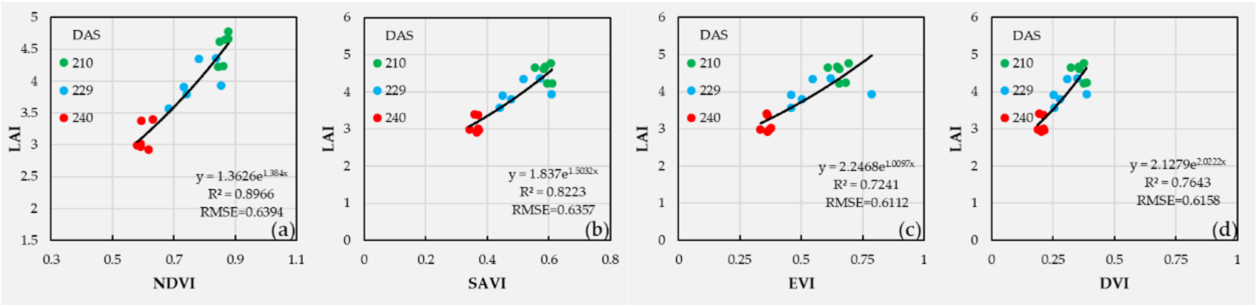

(iii)

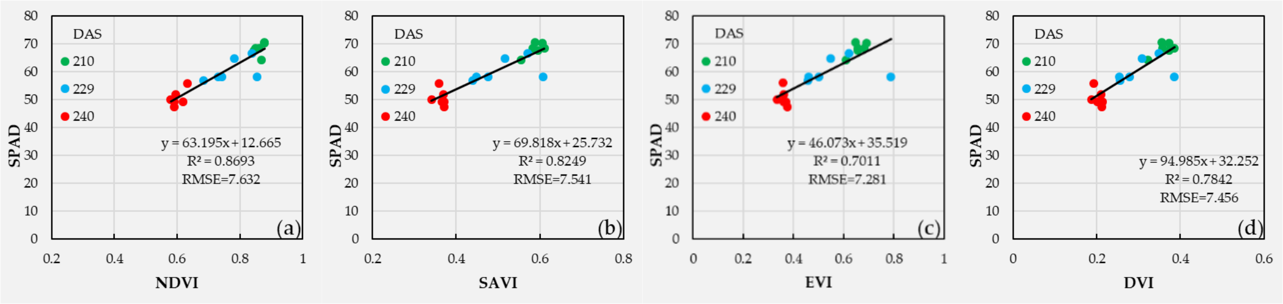

(iv)

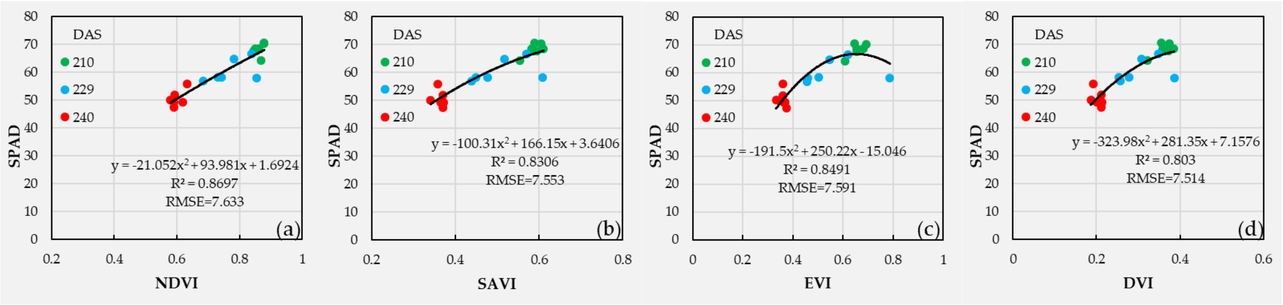

(v)

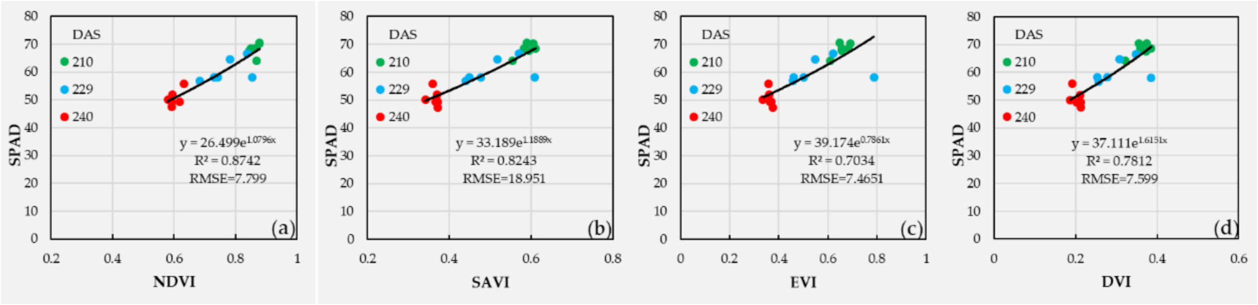

(vi)

Figure A2. LAI and SPAD estimation under Medium water treatment based on VIs; (i) and (iv) were Linear regression model, (ii) and (v) were quadratic polynomial regression, (iii) and (vi) were exponential model.

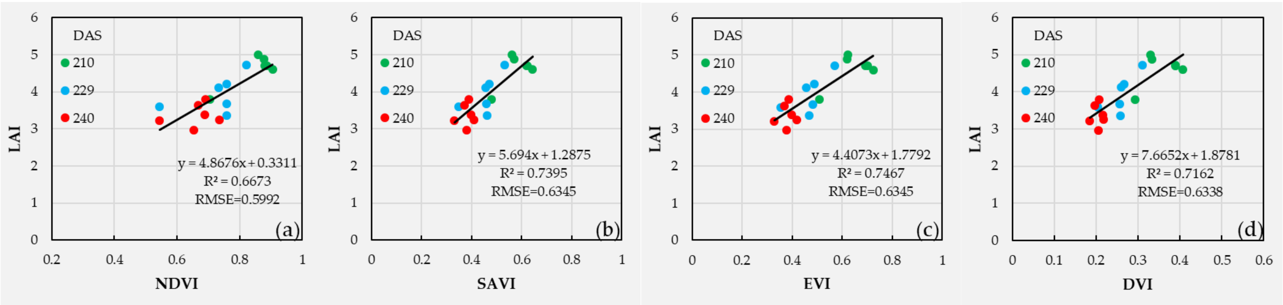

DAS
210
229
240
y = 4.8676x + 0.3311
R^2 = 0.6673
RMSE=0.5992
(a)
NDVI
LAI
y = 5.694x + 1.2875
R^2 = 0.7395
RMSE=0.6345
(b)
SAVI
y = 4.4073x + 1.7792
R^2 = 0.7467
RMSE=0.6345
(c)
EVI
y = 7.6652x + 1.8781
R^2 = 0.7162
RMSE=0.6338
(d)
DVI

(i)

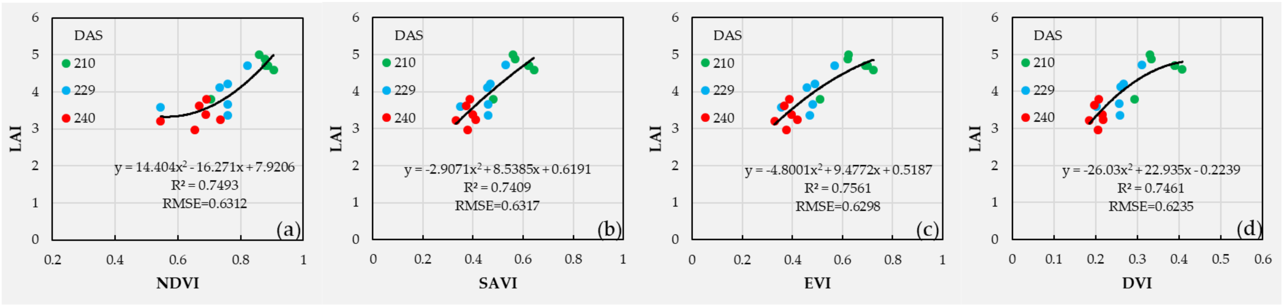

DAS
210
229
240
y = $14.404x^2$ - 16.271x + 7.9206
R^2 = 0.7493
RMSE=0.6312
(a)
NDVI
LAI
y = $-2.9071x^2$ + 8.5385x + 0.6191
R^2 = 0.7409
RMSE=0.6317
(b)
SAVI
y = $-4.8001x^2$ + 9.4772x + 0.5187
R^2 = 0.7561
RMSE=0.6298
(c)
EVI
y = $-26.03x^2$ + 22.935x - 0.2239
R^2 = 0.7461
RMSE=0.6235
(d)
DVI

(ii)

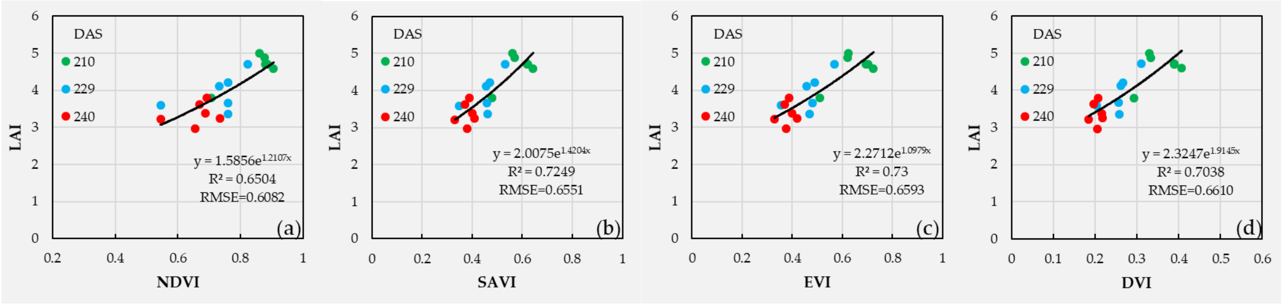

DAS
210
229
240
y = $1.5856e^{1.2107x}$
R^2 = 0.6504
RMSE=0.6082
(a)
NDVI
LAI
y = $2.0075e^{1.4204x}$
R^2 = 0.7249
RMSE=0.6551
(b)
SAVI
y = $2.2712e^{1.0979x}$
R^2 = 0.73
RMSE=0.6593
(c)
EVI
y = $2.3247e^{1.9145x}$
R^2 = 0.7038
RMSE=0.6610
(d)
DVI

(iii)

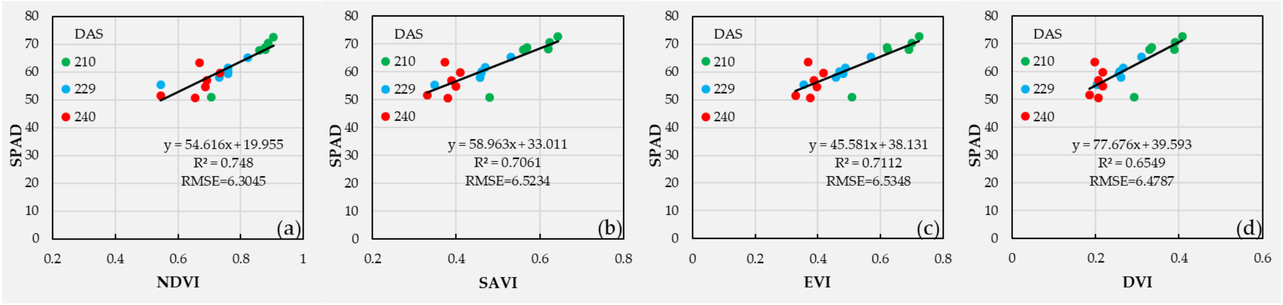

DAS
210
229
240
y = 54.616x + 19.955
R^2 = 0.748
RMSE=6.3045
(a)
NDVI
SPAD
y = 58.963x + 33.011
R^2 = 0.7061
RMSE=6.5234
(b)
SAVI
y = 45.581x + 38.131
R^2 = 0.7112
RMSE=6.5348
(c)
EVI
y = 77.676x + 39.593
R^2 = 0.6549
RMSE=6.4787
(d)
DVI

(iv)

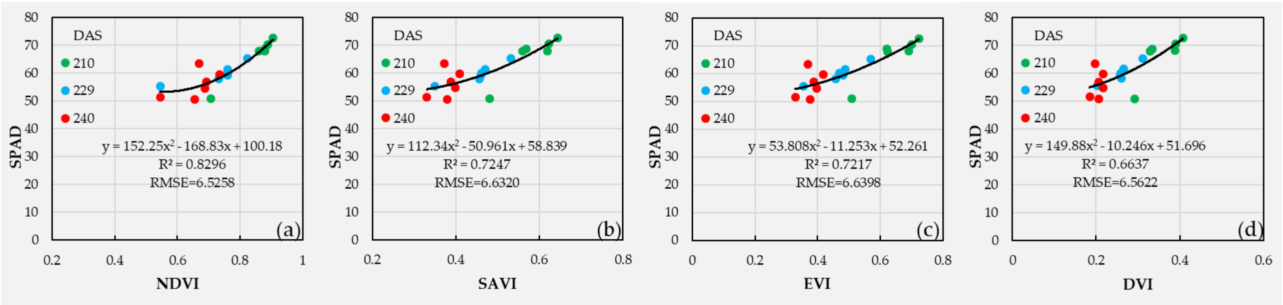

(v)

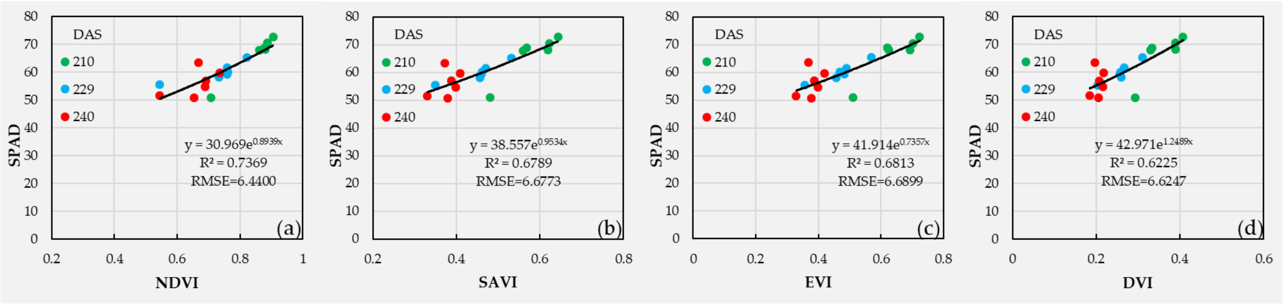

(vi)

Figure A3. LAI and SPAD estimation under High water treatment based on VIs; (i) and (iv) were Linear regression model, (ii) and (v) were quadratic polynomial regression, (iii) and (vi) were exponential model.
